# Photodynamic therapy versus anti-vascular endothelial growth factor agents for polypoidal choroidal vasculopathy: A meta-analysis

**DOI:** 10.1186/s12886-015-0064-5

**Published:** 2015-07-25

**Authors:** Meng Yong, Minwen Zhou, Guohua Deng

**Affiliations:** Division of Opthalmology, Third People’s Hospital of Changzhou, Changzhou, China; Department of Ophthalmology, Shanghai First People’s Hospital, School of Medicine, Shanghai JiaoTong University, Shanghai, China

**Keywords:** polypoidal choroidal vasculopathy, photodynamic therapy, anti- vascular endothelial growth factor

## Abstract

**Background:**

The aim of this study was to evaluate the efficacy and tolerability of photodynamic therapy (PDT) compared to intravitreal vascular endothelial growth factor (VEGF) inhibitors in the treatment of polypoidal choroidal vasculopathy (PCV).

**Methods:**

Relevant studies were selected through an extensive search of the PubMed, EMBASE, Web of Science, and Cochrane Library databases. Outcomes of interest included visual outcomes, anatomic variables, and adverse events.

**Results:**

Six studies enrolling a total of 346 patients were included. The weighted mean differences (WMDs) of the mean changes in LogMAR VA when comparing PDT with anti-VEGF were −0.02 (95 % confidence interval [CI]: −0.12–0.08) at 3 months, 0.02 (95 % CI: −0.12–0.16) at 6 months, 0.02 (95 % CI: −0.15–0.18) at 12 months, and −0.17 (95 % CI: −0.90–0.55) at 24 months. There were no significant differences between the two groups at any of the time points. PDT was found to be associated with greater reduction of central retinal thickness (CRT) at six months (WMD: 44.94; 95 % CI: 16.44–73.44; P = 0.002), and it was superior to anti-VEGF therapy in achieving complete polyp regression (odd ratio, OR: 6.85; 95 % CI: 2.15–21.79; P = 0.001).Rates of adverse events did not differ significantly between the two treatments.

**Conclusions:**

PDT appeared to result in greater CRT reduction at six months and higher polyp regression rate. However, the two treatments appear to be comparable in terms of best corrected visual acuity change and adverse events.

## Background

Polypoidal choroidal vasculopathy (PCV) is a distinct choroidal abnormality characterized by an abnormal branching vascular network in polypoidal structures [1]. It usually causes a decrease in vision through its serious complications such as subretinal hemorrhage, macular edema, and retinal pigment epithelial detachment [2]. Different forms of PCV have different prognoses. The natural course of PCV, which is characterized by clustered polypoidal choroidal lesions, is always related to a poor prognosis [3]. Several studies have reported that PCV is more prevalent in Japanese and other Asian populations than in Caucasian populations [4–6].

Regarding the treatment modalities for PCV, photodynamic therapy (PDT) has been widely used and has shown encouraging results in the regression of polypoidal lesions and stabilization or improvement of visual acuity [7–9]. However, recurrent or newly developed polypoidal lesions may negatively affect the efficacy of this treatment method [10, 11].

Anti-vascular endothelial growth factor (VEGF) therapy is another treatment modality for PCV that is being investigated [12–14]. Intravitreal injection of anti-VEGF agents, including bevacizumab [15] and ranibizumab [16], were tested against PDT and found to reduce exudative fluid, but they did not affect the original abnormal vasculatures [17].

The optimum treatment for PCV remains controversial. To date, a number of studies have compared the anatomic and functional outcomes of PDT and intravitreal injection of anti-VEGF agents for treating PCV [7, 18–22]. However, most are studies with small sample sizes, and no definitive conclusions regarding objective differences in outcomes have been reached. Some of the studies found that PDT resulted in a significantly better outcome than anti-VEGF treatment [7, 22], whereas other studies reported different results [19, 20]. Therefore, we performed a systematic review and meta-analysis based on relevant published controlled clinical trials to evaluate whether PDT offers any advantages over anti-VEGF in terms of anatomic and functional outcomes when treating PCV.

## Methods

This meta-analysis was performed according to a predetermined protocol, described below. In addition, standard systematic review guidelines outlined by the Cochrane Handbook for Systematic Reviews of Interventions and Preferred Items for Systematic Reviews and Meta-Analysis (PRISMA) Statement were followed [23].

### Literature search

A literature search of PubMed, ISI Web of Science, EMBASE, and the Cochrane library was performed to identify relevant studies. The following terms were used for the searches: (“polypoidal choroidal vasculopathy” OR PCV) AND (“angiogenesis inhibitors” OR “endothelial growth factors” OR VEGF OR lucentis OR ranibizumab OR bevacizumab OR avastin) AND (“photodynamic therapy” OR PDT). The websites of professional associations and Google Scholar were also searched for additional information. Once relevant articles were identified, their reference lists were searched for additional articles. The final search was carried out in March 2014, without restrictions regarding publication year, language, or methodological filter.

### Inclusion and exclusion criteria

The following inclusion criteria were used: (i) study type: comparative studies; (ii) population: treatment-naive patients with PCV; (iii) intervention: PDT alone versus anti-VEGF alone; and (iv) outcome variables: evaluation of at least one of the outcomes of interest mentioned below. Editorials, letters to the editor, review articles, case reports, meeting abstracts, and animal experimental studies were excluded.

### Outcome measures

The following outcomes were used to compare PDT and anti-VEGF. (1) Visual outcomes: mean visual acuity (VA) change at three, six, 12, and 24 months; and proportion of eyes with improved, stable, and deteriorated vision at endpoint. After assessing VA at each follow up visit, the patients were categorized into three groups based on their VA change from baseline: improved, stable, and deteriorated VA. The VA was considered to be improved or deteriorated when the change in the logMAR VA exceeded 0.3 units. (2) Anatomical outcomes: mean change in central retinal thickness (CRT) at three, six, and 12 months and regression rates of polyps. (3) Adverse events: incidence of retinal hemorrhage.

### Data extraction

Two reviewers (Z.M.W. and M.Y.) independently extracted data from the included studies, and disagreements were resolved by discussion until a consensus was reached. The following information was extracted from each study: first author; year of publication; study design; location of the trial, follow up; baseline patient characteristics; inclusion and exclusion criteria; and outcomes of interest. Patients reporting adverse events were also recorded.

### Assessment of methodology quality

The qualities of the clinical trials included were assessed by two independent observers (Z.M.W. and M.Y.) using a previously reported quality assessment system for both randomized and nonrandomized studies [24]. The system includes 27 items distributed among five subscales: reporting (ten items), external validity (three items), bias (seven items), confounding (six items), and power (one item). Any discrepancy in qualitative assessment between the two observers was discussed, and a consensus was reached. The total score for each trial was expressed as a percentage of the maximum achievable score. A score not lower than 50 % is considered good quality.

### Statistical analysis

Data from this meta-analysis are presented in accordance with PRISMA guidelines [25]. Weighted mean differences (WMDs) and odds ratios (ORs) were used to compare continuous and dichotomous variables, respectively. All outcomes were reported with 95 % confidence interval (CI). Considering the different clinical characteristics among study groups and the different sample sizes, we assumed that heterogeneity was present even when no statistical significance was identified, and we decided to combine data by using a random effects model to achieve more conservative estimates [26]. Statistical heterogeneity between studies was assessed using the chi-squared test, and the quantity of heterogeneity was evaluated using the I^2^ statistic. A *p* value <0.05 was considered statistically significant. All statistical analyses were performed using Stata (version 12; StataCorp, College Station, TX).

### Sensitivity analysis and publication bias

A sensitivity analysis was undertaken to evaluate the effect of the methodological characteristics of controlled clinical trials in terms of trial design and different anti-VEGF agents. Potential publication bias was evaluated with Begg’s and Egger’s tests [27, 28].

## Results

### Literature search

**Fig. 1:**
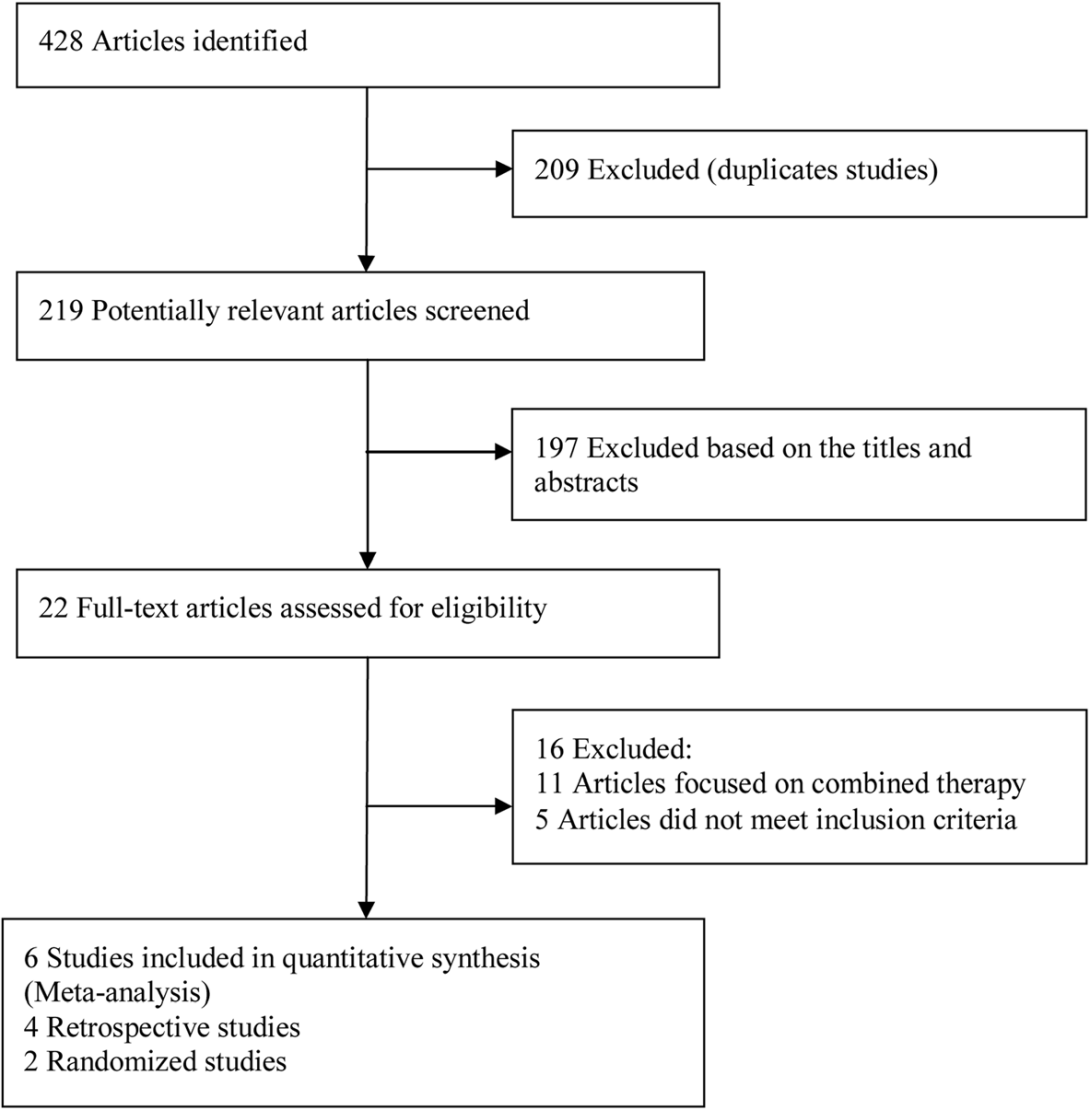
Flowchart of publication search and selection

A total of 428 papers were identified by our literature search, of which 209 were excluded as duplicate studies and 197 were excluded based on the titles and abstracts. The remaining 22 studies were retrieved for full-text review. Eleven of the studies were excluded because they focused on combined therapy, three case reports were excluded, and two articles were excluded because they included non-treatment-naive patients. Thus, a final total of six studies published between 2010 and 2013 were included in this meta-analysis [7, 18–22]. The trial selection process is shown in Fig. 1.

### Study characteristics and quality

The characteristics of the included studies are shown in Tables 1 and 2. A total of 346 eyes of 346 patients were enrolled, with the mean age ranging from 62.2 to 75.4 years. The duration of the studies ranged from three to 24 months. Two trials had a prospective, parallel, randomized design, and four had a retrospective, nonrandomized design. The quality assessment is summarized in Table 3. The Downs and Black scores of all of the studies were over 16 (50 %), and the scores of both of the randomized clinical trials (RCTs) were over 24 (75 %).

**Table 1:** Characteristics of included studies

First Author (year)	Design	Center	Location	Type of OCT	PDT	anti-VEGF	Sex(male/female)		Age^a ^(year)	Follow- up^a^(mo)
					No. eyes/patients	No. eyes/patients	PDT	anti-VEGF		
Mitamura(2010)^22^	Retro	1	Japan	TD-OCT	49/49	22/22	7/42	5/17	73.0/69.6	3/3
Rouvas (2011)^7^	Retro	2	Greece	SD-OCT	11/11	10/10	6/5	5/5	62.9/66.5	12/12
Koh (2012)^21^	RCT	7	Hong Kong, Singapore, Korea, Taiwan, Thailand	TD-OCT	21/21	21/21	15/6	15/6	62.2/69.3	6/6
Oishi (2013)^19^	RCT	5	Japan	SD-OCT	47/47	46/46	32/15	28/18	75.0/75.4	12/12
Inoue (2013)^20^	Retro	1	Japan	SD-OCT	44/44	33/33	30/14	19/14	71.0/73.2	24/24
Kang (2014)^18^	Retro	1	Korea	TD-OCT	19/19	23/23	NA	NA	66.2/68.0	24/24

^a^PDT group/anti-VEGF group

*PDT* photodynamic therapy; *VEGF* vascular endothelial growth factor; *mo* months; *Retro* retrospective comparative study; *TD-OCT* time-domain optical coherence tomography; *SD-OCT* spectral-domain optical coherence tomography; *RCT* prospective randomized controlled

**Table 2:** Characteristics of lesions and treatment exposures included in the meta-analysis

Study	Group	Lesion GLD (μm) (mean ± SD)	Interventions	Number of Treatments (mean ± SD) (range)	Follow up duration (mo)	Diagnosis of PCV
Mitamura (2010)	PDT	3718 ± 1665	PDT (6 mg/m^2^)	1PDT	3	Presence of reddish-orange lesions; recurrent serosanguinous RPE detachments; dilated network of inner choroidal vessels with terminal hyperfluorescent aneurysm-like dilatations (polyps) on ICGA.
	Anti-VEGF	3651 ± 1833	IVB 1.25 mg	3IVB	3	
Rouvas (2011)	PDT	NA	PDT (6 mg/m^2^)	1.82(1–3)PDT	12	Identification of polyps and interconnecting vessels on the ICGA; presence of subretinal hemorrhages and/or exudation in the macula based on clinical examination
	Anti-VEGF	NA	IVR 0.5 mg	6.9 (3–11)IVR	12	
Oishi (2013)	PDT	3051.1 ± 1177.7	PDT (6 mg/m^2^)	1.8PDT	12	PCV was diagnosed based on the presence of polypoidal lesion depicted with ICGA
	Anti-VEGF	3347.4 ± 1288.3	IVR 0.5 mg	2.5 IVR	12	
Koh (2012)	PDT	<5400	PDT (6 mg/m^2^) + sham	1.7(1–4) PDT	6	Presence of early subretinal focal ICGA hyperfluorescence; at least one of the following clinical criteria: presence of pulsatile polyp; presence of hypofluorescent halo; orange subretinal nodules in fundus photograph
	Anti-VEGF	<5400	IVR 0.5 mg + sham	5.2 (3–6)IVR	6	
Inoue (2013)	PDT	3640 ± 2120	PDT (6 mg/m^2^)	1.52 ± 0.66 PDT	24	presence of clinical, OCT, FA and confocal ICGA findings showing a branching vascular network and polypoidal structures
	Anti-VEGF	4171 ± 2631	IVR 0.5 mg	7.1 ± 5.2 IVR	24	
Kang (2014)	PDT	2810.87 ± 974.10	PDT (6 mg/m^2^)	2.56 ± 0.38 PDT	24	PCV with subfoveal leakage on FA; presence of branching vascular networks and polypoidal lesions on ICGA
	Anti-VEGF	2790.05 ± 871.50	IVR 0.5 mg or IVB 1.25 mg	10.12 ± 1.46 IVR/IVB	24	

*GLD* greatest linear dimension; *SD* standard deviation; *PCV* polypoidal choroidal vasculopathy; *PDT* photodynamic alone; *RPE* retinal pigment epithelium; *VEGF* vascular endothelial growth factor; *IVB* intravitreal bevacizumab; *ICGA* indocyanine green angiography; *NA* not available; *IVR* intravitreal ranibizumab; *OCT* optical coherence tomography; *FA* fluorescein angiography

**Table 3:** Quality scoring components for six clinical trials included

	Quality score component					Score	
First Author(year)	I	II	III	IV	V	Over all	Percentage
Mitamura(2010)	8	2	4	2	1	17	53.13 %
Rouvas (2011)	9	2	4	2	1	18	56.25 %
Koh (2012)	11	3	5	3	2	24	75.00 %
Oishi (2013)	11	3	5	5	2	26	81.25 %
Inoue (2013)	9	2	4	2	2	19	59.38 %
Kang (2014)	9	2	4	3	2	20	62.50 %

### Visual outcomes

VA was the most important criterion for evaluating efficacy. Differences in mean LogMAR VA changes between the two groups are presented in Table 4. No significant differences in BCVA change were found in the PDT group compared with the anti-VEGF group at three months (WMD, −0.02; 95 % CI, −0.12–0.08); six months (WMD, 0.02; 95 % CI, −0.12–0.16); 12 months (WMD, 0.02; 95 % CI, −0.15–0.18), and 24 months (WMD, −0.17; 95 % CI, −0.90–0.55) post-treatment. Substantial statistical heterogeneity was observed across studies at the 6-, 12- and 24-month time points. We then divided the studies into subgroups according to study design (retrospective and randomized) and different anti-VEGF agents. There were no statistically significant differences in mean BCVA changes between the PDT group and the anti-VEGF group at all subgroups, with the exception of one RCT subgroup at the 12-month time point. When VA change was treated as a categorical variable, the percentages of improved, stable, and deteriorated VA at final visits were compared. The rates of improved, stable, and deteriorated BCVA were comparable between the two groups (Table 4).

**Table 4:** Pooled estimates for BCVA change from baseline for PDT versus anti-VEGF

Outcome of Interest	Studies (n)	WMD/OR (95 % CI)	Test for Overall Effect	Study Heterogeneity		
				χ^2^	*p*	I^2^
LogMAR Change in both Groups (PDT group vs anti-VEGF group) (3mo)						
Design						
All trials	5	−0.02 (−0.12, 0.08)	Z =0.45, *P* =0.653	6.77	0.148	40.9 %
Retro	4	−0.05 (−0.18, 0.07)	Z =0.85, *P* =0.407	5.10	0.165	41.2 %
RCT	1	0.05 (−0.07, 0.17)	Z =0.83, *P* =0.407		-	
Anti-VEGF agents						
All trials	5	−0.02 (−0.12, 0.08)	Z =0.45, *P* =0.653	6.77	0.148	40.9 %
Ranibizumab	3	−0.03 (−0.20, 0.13)	Z =0.39, *P* =0.694	6.71	0.035	70.2 %
Non- Ranibizumab	2	−0.02 (−0.16, 0.12)	Z =0.32, *P* =0.749	0.02	0.859	0.00 %
LogMAR Change in both Groups (PDT group vs anti-VEGF group) (6mo)						
Design						
All trials	4	0.02 (−0.12, 0.16)	Z =0.23, *P* =0.817	7.60	0.055	60.5 %
Retro	3	−0.03 (−0.22, 0.17)	Z =0.25, *P* =0.800	5.74	0.057	65.2 %
RCT	1	0.10 (−0.02, 0.22)	Z =1.66, *P* =0.097		-	
Anti-VEGF agents						
All trials	4	0.02 (−0.12, 0.16)	Z =0.23, *P* =0.817	7.60	0.055	60.5 %
Ranibizumab	3	0.02 (−0.15, 0.20)	Z =0.27, *P* =0.787	7.21	0.027	72.3 %
Non- Ranibizumab	1	−0.03 (−0.27, 0.21)	Z =0.25, *P* =0.806		-	
LogMAR Improvements in both Groups (PDT group vs anti-VEGF group) (12mo)						
Design						
All trials	4	0.02 (−0.15, 0.18)	Z =0.20, *P* =0.839	10.43	0.015	71.2 %
Retro	3	−0.04 (−0.24, 0.16)	Z =0.40, *P* =0.690	5.99	0.050	66.6 %
RCT	1	0.15 (0.03, 0.27)	Z =2.49, *P* =0.013		-	
Anti-VEGF agents						
All trials	4	0.02 (−0.15, 0.18)	Z =0.20, *P* =0.839	10.43	0.015	71.2 %
Ranibizumab	3	0.03 (−0.17, 0.24)	Z =0.31, *P* =0.760	9.63	0.009	79.0 %
Non- Ranibizumab	1	−0.05 (−0.29, 0.19)	Z =0.41, *P* =0.682		-	
LogMAR Improvements in both Groups (PDT group vs anti-VEGF group) (24mo)						
All trials	2	−0.17 (−0.90, 0.55)	*Z* =0.47, *P* =0.638	19.1	*P* < 0.001	94.8 %
LogMAR Change as Categorical Variable						
Proportion of eyes with improved vision						
final visit	5	1.24 (0.54, 2.85)	*Z* =0.51, *P* =0.610	7.47	0.113	46.4 %
Proportion of eyes with deteriorated vision						
final visit	5	1.40 (0.42, 4.73)	*Z* =0.55, *P* =0.586	11.23	0.024	64.4 %
Proportion of eyes with stable vision						
final visit	5	0.56 (0.29, 1.10)	*Z* =1.67, *P* =0.094	6.82	0.145	41.4 %

*PDT* photodynamic therapy; *VEGF* vascular endothelial growth factor; *WMD* weighted mean differences; *OR* odds ratio; *CI* confidence interval; *Retro* retrospective comparative study; *RCT* prospective randomized controlled trial

### Central retinal thickness

CRT was defined as the distance between the internal limiting membrane and the inner surface of the RPE, and measured manually at the fovea. CRT was reported as the mean change from baseline to follow up month and was measured by optical coherence tomography (OCT). The pooled results revealed that at the six-month follow up, change in CRT was significantly higher in the PDT group than in the anti-VEGF group (WMD, 44.94; 95 % CI, 16.44–73.44). However, this difference was not statistically significant at three and 12 months, with WMDs of 18.69 (95 % CI: −0.83–38.20) and 13.91(95 % CI: −42.14–69.97), respectively. No substantial statistical heterogeneity was observed across studies at most time points, with the exception of the12-month time point. We also divided the studies into subgroups according to study design (retrospective and randomized). The subgroup analysis indicated that none of the subgroups materially altered the pooled results. Stratification by different type of OCT also showed that different type of OCT did not alter the pooled results at any follow up time point (Table 5).

**Table 5:** Pooled estimates for CRT reduction from baseline for PDT versus anti-VEGF

Outcome of interest	Studies (n)	WMD (95 % CI)	Test for Overall Effect	Study Heterogeneity		
				χ^2^	*p*	I^2^
CRT Reduction (3mo)						
Design						
All trials	5	18.69 (−0.83, 38.20)	Z =1.88, *P* =0.061	2.36	0.670	0.00 %
Retro	3	22.75 (−0.96, 44.54)	Z =1.05, *P* =0.141	1.56	0.459	0.00 %
RCT	2	2.24 (−41.62, 46.09)	Z =0.10, *P* =0.920	0.13	0.719	0.00 %
Type of OCT						
TD-OCT	3	10.47 (−30.90, 51.85)	Z =0.50, P =0.620	1.29	0.526	0.00 %
SD-OCT	2	21.03(−1.09, 43.17)	Z =1.86, P =0.062	0.88	0.348	0.00 %
CRT Reduction (6mo)						
Design						
All trials	4	44.94 (16.44, 73.44)	Z =3.09, *P* =0.002	4.30	0.231	30.3 %
Retro	2	36.87 (14.58, 59.16)	Z =3.24, *P* =0.001	0.65	0.421	0.00 %
RCT	2	66.62 (3.42, 129.81)	Z =2.07, *P* =0.039	2.05	0.152	51.3 %
Type of OCT						
TD-OCT	2	22.15 (2.98, 67.30)	Z =2.06, P =0.038	0.17	0.678	0.00 %
SD-OCT	2	62.13 (8.19, 116.07)	Z =2.26, P =0.024	3.08	0.079	67.5 %
CRT Reduction (12mo)						
Design						
All trials	3	13.91(−42.14, 69.97)	Z =0.49, *P* =0.627	8.48	0.014	76.4 %
Retro	2	36.03 (−13.52, 85.58)	Z =1.43, *P* =0.154	2.48	0.115	59.6 %
RCT	1	13.91 (−42.14, 69.97)	Z =1.00, *P* =0.315	-	-	
Type of OCT						
TD-OCT	1	1.35 (−60.65, 63.35)	Z =0.04, P =0.966	-	-	-
SD-OCT	2	16.70 (−65.61, 99.00)	Z =0.40, P =0.691	6.92	0.009	85.6 %

*CRT* central retinal thickness; *PDT* photodynamic therapy; *VEGF* vascular endothelial growth factor; *WMD* weighted mean differences; *CI* confidence interval; *Retro* retrospective comparative study; *RCT* prospective randomized controlled trial; *OCT* optical coherence tomography; *TD-OCT* time-domain optical coherence tomography; *SD-OCT* spectral-domain optical coherence tomography

### Regression rates of polyps and adverse events

**Fig. 2:**
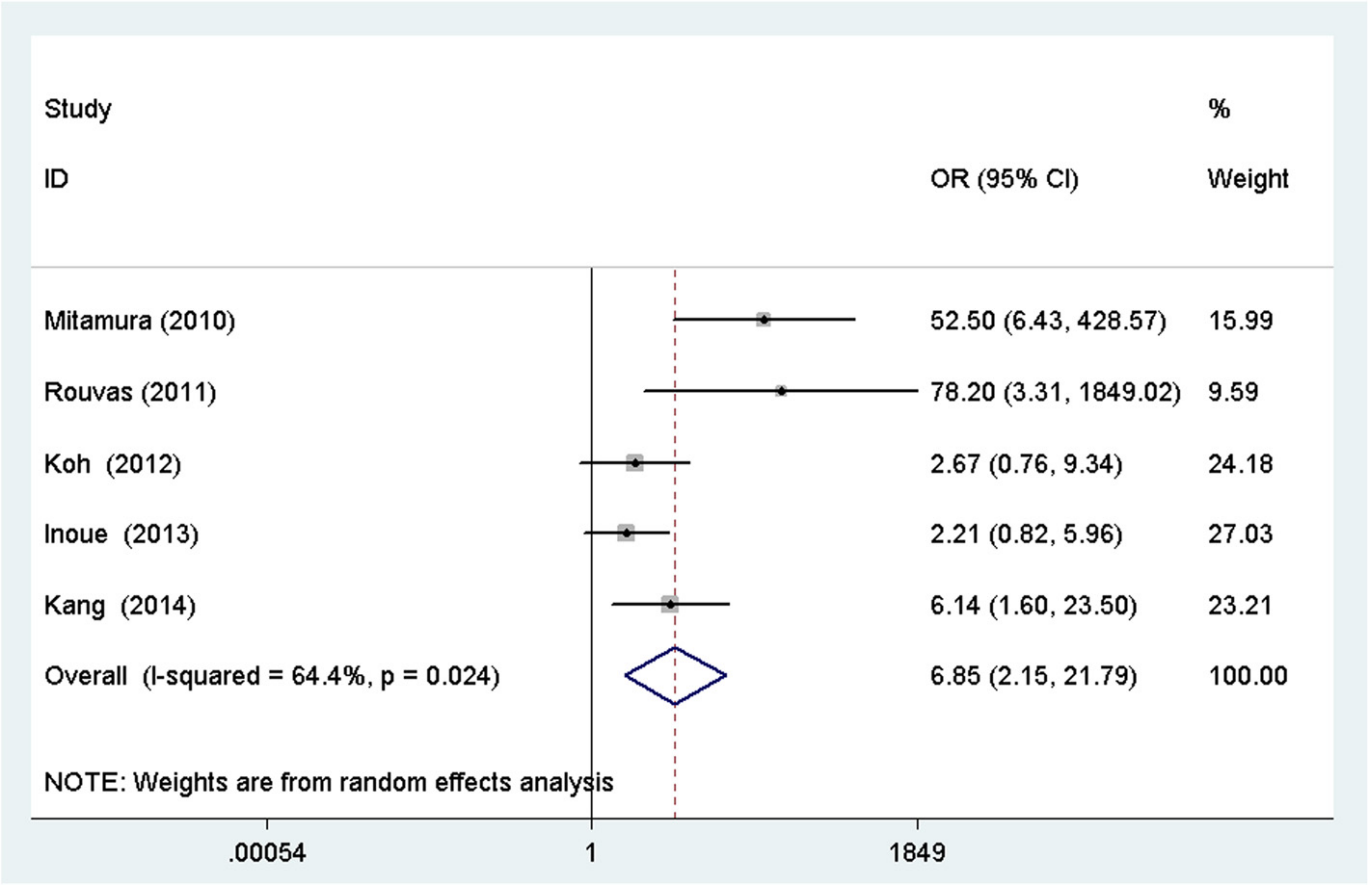
Forest plot of the risk estimates of the complete polyp regression rate between PDT and anti-VEGF

Five studies reported the regression rates of polyps at the follow up end point, and PDT was superior to anti-VEGF therapy in achieving complete polyp regression (OR: 6.85; 95 % CI: 2.15–21.79; *p* =0.001) (Fig. 2). There were insufficient data about adverse effects, therefore, most of adverse effects were not pooled in the present meta-analysis. Retinal hemorrhage was the most common complication; the pooled data showed no significant difference between the two groups (OR: 2.44; 95 % CI: 0.83–7.1; *p* = 0.104).

### Publication bias

Assessment of publication bias using Egger’s and Begg’s tests based on mean changes in LogMAR VA at three months showed that there was little potential publication bias among the included trials (Egger’s test, *p* = 0.327; Begg’s test, *p* =0.221).

## Discussion

PCV is a choroidal vascular disorder that can be treated effectively by PDT or anti-VEGF therapy. For many years, the scientific debate regarding the comparative effectiveness of PDT and anti-VEGF treatment for PCV has remained unsettled. In the present meta-analysis, we reviewed the literature on the efficacy and safety of PDT and anti-VEGF treatment in the management of PCV. The results of this systematic review showed that PDT was more effective in CRT reduction than anti-VEGF at six months post-treatment and that PDT was more effective than anti-VEGF in achieving regression of polyps. However, these two treatment procedures appear to be equivalent in terms of BCVA change at all follow up time points. It should be noted that the frequency of retreatments for both PDT and anti-VEGF groups is not uniform in different studies. This might be a confounder between the study groups. However, the results from our subgroup analyses were quite similar and robust. None of the outcome results were significantly modified by research design or different anti-VEGF agents used, except one subgroup, which included only one RCT trial.

VA, a primary measure of treatment efficacy, is an exceedingly important outcome. Our meta-analysis of all changes in logMAR BCVA during the follow up months revealed that PDT was comparable with anti-VEGF therapy at all follow up time points. The direct effect of PDT on polypoidal lesions and the subsequent resolution of exudative fluid might lead to a favorable visual outcome. As for anti-VEGF, the rapid resolution of exudative fluid and retinal edema might be the cause of the favorable BCVA. CRT is another strong prognostic measure of PCV severity. At six months, significant decreases in CRT were found in the PDT group compared with the anti-VEGF group. The fact that PDT induces more CRT reduction, but not more BCVA change, than anti-VEGF confirmed the notion that reduction of CRT does not necessarily indicate a good visual outcome.

A number of studies have shown encouraging results for PDT’s effect on the vascular lesions of PCV, with complete regression of the polypoidal lesions achieved with fewer sessions in many cases [2, 7]. The pooled result of this meta-analysis also showed that PDT resulted in superior efficacy in terms of polyp regression compared to anti-VEGF therapy, which is consistent with most studies. This finding seemed reasonable, because PDT is known to be effective for treating PCV as a result of its photothrombotic effect on choroidal vascular lesions with polyps [2]. On the other hand, anti-VEGF treatment has its own inherent merits of reducing exudative lesions, leakage, resolving fluids, and improving VA, but it is ineffective for polyp regression [7, 21]. In this context, treatment with both PDT to resolve the polypoidal lesions and anti-VEGF agents to reduce the exudative lesions might be an ideal therapy method when treating PCV. The previous meta-analysis demonstrated that a combined-treatment appeared to result in better visual acuity and lower retinal hemorrhage [29].

Several studies have reported that PDT usually has more vision-threatening side effects, such as the induction of severe subretinal hemorrhage, than anti-VEGF therapy [30,31]. Nevertheless, the present meta-analysis showed that the incidence of subretinal hemorrhage complication is comparable between the two treatment methods. Of note, the present meta-analysis only included two studies, the small sample size should also be taken into consideration and this finding should be interpreted with caution.

Substantial heterogeneity was observed among studies when comparing the efficacy of PDT and anti-VEGF, which was not surprising, given the differences in the various matching criteria and non-standardized measurement of outcomes. Using the random-effects model to pool the data might reduce, but will not abolish, the effect of heterogeneity.

Our study has a number of strengths. First, the meta-analysis was a direct comparison between PDT and anti-VEGF, rather than an indirect comparison. Second, the meta-analysis had strict inclusion and exclusion criteria; we excluded two studies that included patients who had previously received PDT or anti-VEGF treatment [32, 33]. Third, we strictly followed the Cochrane Handbook for Systematic Reviews of Interventions and the PRISMA statement, including the literature search, data extraction, quality assessment, and statistical analysis, thereby making our conclusions more scientific and reliable. Thus, this meta-analysis provides the most up-to-date information in this area.

There were some limitations to this study. First, all of the included studies were retrospective, except for two RCTs with small sample sizes. Inadequate random sequence generation and blinding could result in selection bias, as patients with worse visual prognoses might be offered the combination treatment. Nonetheless, the major characteristics of the eyes in the two groups were comparable at baseline, and therefore, selection bias was less likely to occur. Second, the anti-VEGF group used either bevacizumab or ranibizumab as an anti-VEGF agent, and there might be a difference between the two agents when treating PCV. However, subgroup analysis showed that they had similar results. Therefore, the effect of different anti-VEGF agents might not significantly affect the results. The third limitation was the presence of between-studies heterogeneity. A random effects model was used when statistically significant heterogeneity was encountered. Fourth, as we could not gain access to unpublished results, publication bias cannot be fully excluded. A fifth limitation is that the analyses of VA change, which was treated as a categorical variable, regression rates of polyps, and adverse events were based on data pooled from trials of different durations due to a lack of reported data in all follow-up phases. It was a compromise proposal to choose the follow-up end-point data. Lastly, to avoid publication bias, we conducted not only an electronic search, but also a manual search in order to identify all potentially relevant articles, including both published and unpublished studies. Unfortunately, it is possible that we may have failed to include some papers, especially those published in other languages.

## Conclusions

To the best of our knowledge, this is the first systematic review to focus on the question of whether PDT or anti-VEGF treatment is more effective for PCV. The results showed that PDT produced better CRT reduction at six months post-treatment, as well as higher rates of polypoidal regression, than anti-VEGF treatment. Despite these encouraging findings, the inherent limitations of the included studies should be considered, and conclusions drawn from our pooled results should be interpreted with caution. Future large-volume, well-designed RCTs with extensive follow-up are needed to confirm and update the findings of this analysis.

## References

[1] Yannuzzi LA, Sorenson J, Spaide RF, Lipson B (1990). Idiopathic polypoidal choroidal vasculopathy (IPCV). Retina.

[2] Chan WM, Lam DS, Lai TY, Liu DT, Li KK, Yao Y, Wong TH (2004). Photodynamic therapy with verteporfin for symptomatic polypoidal choroidal vasculopathy: one-year results of a prospective case series. Ophthalmology.

[3] Bessho H, Honda S, Imai H, Negi A (2011). Natural course and funduscopic findings of polypoidal choroidal vasculopathy in a Japanese population over 1 year of follow-up. Retina.

[4] Sho K, Takahashi K, Yamada H, Wada M, Nagai Y, Otsuji T, Nishikawa M, Mitsuma Y, Yamazaki Y, Matsumura M, Uyama M (2003). Polypoidal choroidal vasculopathy: incidence, demographic features, and clinical characteristics. Arch Ophthalmol.

[5] Obata R, Yanagi Y, Kami J, Takahashi H, Inoue Y, Tamaki Y (2006). Polypoidal choroidal vasculopathy and retinochoroidal anastomosis in Japanese patients eligible for photodynamic therapy for exudative age-related macular degeneration. Jpn J Ophthalmol.

[6] Hou J, Tao Y, Li XX, Zhao MW (2011). Clinical characteristics of polypoidal choroidal vasculopathy in Chinese patients. Graefes Arch Clin Exp Ophthalmol.

[7] Rouvas AA, Papakostas TD, Ntouraki A, Douvali M, Vergados I, Ladas ID (2011). Photodynamic therapy, ranibizumab, and ranibizumab with photodynamic therapy for the treatment of polypoidal choroidal vasculopathy. Retina.

[8] Sato T, Kishi S, Matsumoto H, Mukai R (2013). Comparisons of outcomes with different intervals between adjunctive ranibizumab and photodynamic therapy for polypoidal choroidal vasculopathy. Am J Ophthalmol.

[9] Silva RM, Figueira J, Cachulo ML, Duarte L, Faria DAJ, Cunha-Vaz JG (2005). Polypoidal choroidal vasculopathy and photodynamic therapy with verteporfin. Graefes Arch Clin Exp Ophthalmol.

[10] Sayanagi K, Gomi F, Sawa M, Ohji M, Tano Y (2007). Long-term follow-up of polypoidal choroidal vasculopathy after photodynamic therapy with verteporfin. Graefes Arch Clin Exp Ophthalmol.

[11] Akaza E, Mori R, Yuzawa M (2008). Long-term results of photodynamic therapy of polypoidal choroidal vasculopathy. Retina.

[12] Cho HJ, Kim HS, Jang YS, Han JI, Lew YJ, Lee TG, Kim CG, Kim JW (2013). Effects of choroidal vascular hyperpermeability on anti-vascular endothelial growth factor treatment for polypoidal choroidal vasculopathy. Am J Ophthalmol.

[13] Kang HM, Koh HJ (2013). Long-term visual outcome and prognostic factors after intravitreal ranibizumab injections for polypoidal choroidal vasculopathy. Am J Ophthalmol.

[14] Sonoda S, Sakamoto T, Otsuka H, Yoshinaga N, Yamashita T, Ki-I Y, Okubo A, Yamashita T, Arimura N (2013). Responsiveness of eyes with polypoidal choroidal vasculopathy with choroidal hyperpermeability to intravitreal ranibizumab. BMC Ophthalmol.

[15] Song JH, Byeon SH, Lee SC, Koh HJ, Kwon OW (2009). Short-term safety and efficacy of a single intravitreal bevacizumab injection for the management of polypoidal choroidal vasculopathy. Ophthalmologica.

[16] Mori R, Yuzawa M, Akaza E, Haruyama M (2013). Treatment results at 1 year of ranibizumab therapy for polypoidal choroidal vasculopathy in eyes with good visual acuity. Jpn J Ophthalmol.

[17] Lai TY, Chan WM, Liu DT, Luk FO, Lam DS (2008). Intravitreal bevacizumab (Avastin) with or without photodynamic therapy for the treatment of polypoidal choroidal vasculopathy. Br J Ophthalmol.

[18] Kang HM, Koh HJ (2014). Two-year outcome after combination therapy for polypoidal choroidal vasculopathy: comparison with photodynamic monotherapy and anti-vascular endothelial growth factor monotherapy. Ophthalmologica.

[19] Oishi A, Kojima H, Mandai M, Honda S, Matsuoka T, Oh H, Kita M, Nagai T, Fujihara M, Bessho N (2013). Comparison of the effect of ranibizumab and verteporfin for polypoidal choroidal vasculopathy: 12-month LAPTOP study results. Am J Ophthalmol.

[20] Inoue M, Arakawa A, Yamane S, Kadonosono K (2013). Long-term outcome of intravitreal ranibizumab treatment, compared with photodynamic therapy, in patients with polypoidal choroidal vasculopathy. Eye (Lond).

[21] Koh A, Lee WK, Chen LJ, Chen SJ, Hashad Y, Kim H, Lai TY, Pilz S, Ruamviboonsuk P, Tokaji E (2012). EVEREST study: efficacy and safety of verteporfin photodynamic therapy in combination with ranibizumab or alone versus ranibizumab monotherapy in patients with symptomatic macular polypoidal choroidal vasculopathy. Retina.

[22] Mitamura Y, Kitahashi M, Kubota-Taniai M, Yamamoto S (2010). Comparison of intravitreal bevacizumab to photodynamic therapy for polypoidal choroidal vasculopathy: short-term results. Indian J Ophthalmol.

[23] Higgins JGS (2011). Cochrane Handbook for Systematic Reviews of Interventions Version 5.1.0.

[24] Downs SH, Black N (1998). The feasibility of creating a checklist for the assessment of the methodological quality both of randomised and non-randomised studies of health care interventions. J Epidemiol Community Health.

[25] Moher D, Liberati A, Tetzlaff J, Altman DG (2009). Preferred reporting items for systematic reviews and meta-analyses: the PRISMA statement. J Clin Epidemiol.

[26] DerSimonian R, Laird N (1986). Meta-analysis in clinical trials. Control Clin Trials.

[27] Egger M, Davey SG, Schneider M, Minder C (1997). Bias in meta-analysis detected by a simple, graphical test. BMJ.

[28] Begg CB, Mazumdar M (1994). Operating characteristics of a rank correlation test for publication bias. Biometrics.

[29] Wang W, He M, Zhang X (2014). Combined intravitreal anti-VEGF and photodynamic therapy versus photodynamic monotherapy for polypoidal choroidal vasculopathy: a systematic review and meta-analysis of comparative studies. PLoS One.

[30] Hirami Y, Tsujikawa A, Otani A, Yodoi Y, Aikawa H, Mandai M, Yoshimura N (2007). Hemorrhagic complications after photodynamic therapy for polypoidal choroidal vasculopathy. Retina.

[31] Lee YA, Yang CH, Yang CM, Ho TC, Lin CP, Huang JS, Chen MS (2012). Photodynamic therapy with or without intravitreal bevacizumab for polypoidal choroidal vasculopathy: two years of follow-up. Am J Ophthalmol.

[32] Lai TY, Lee GK, Luk FO, Lam DS (2011). Intravitreal ranibizumab with or without photodynamic therapy for the treatment of symptomatic polypoidal choroidal vasculopathy. Retina.

[33] Saito M, Iida T, Kano M (2011). Intravitreal ranibizumab for polypoidal choroidal vasculopathy with recurrent or residual exudation. Retina.

